# Supplementary material for: A Likelihood Approach for Real-Time Calibration of Stochastic Compartmental Epidemic Models
Source: PLoS Comput Biol. 2017 Jan 17;13(1):e1005257. doi: 10.1371/journal.pcbi.1005257 (PMC5240920; doi:10.1371/journal.pcbi.1005257)
Supplement: S1 File — (TAR.GZ) [file pcbi.1005257.s014.tar.gz › HSPH_Online-SI-Revision/PF08/n10-mild/n10-mild_PF2_table-8.pdf]

1	newly 3 weeks spec	108.	238.46	{188., 290.}	120.796	0.334526	newly 3 weeks cumul	274.	636.78	{519., 767.}	132.401	0.358349	newly 5 weeks spec	177.	274.82	{213., 333.}	55.2655	0.180954	newly 5 weeks cumul	569.	1168.64	{938., 1416.}	105.385	0.303948	newly 58 weeks cumul	3351.	5631.91	{5223., 6244.}	68.0665	0.224374
2	newly 3 weeks spec	133.	230.2	{119., 442.}	79.188	0.209197	newly 3 weeks cumul	288.	557.77	{297., 996.}	95.4132	0.245297	newly 5 weeks spec	307.	315.03	{142., 647.}	46.3355	0.196317	newly 5 weeks cumul	831.	1145.6	{576., 2220.}	54.852	0.169799	newly 63 weeks cumul	4314.	6485.32	{4966., 7846.}	50.5865	0.171573
3	newly 3 weeks spec	119.	300.97	{58., 683.}	171.807	0.369653	newly 3 weeks cumul	252.	657.72	{151., 1287.}	175.151	0.375751	newly 5 weeks spec	232.	461.69	{66., 1100.}	129.685	0.356631	newly 5 weeks cumul	651.	1512.55	{305., 3319.}	152.312	0.357213	newly 66 weeks cumul	4852.	6675.29	{3761., 8970.}	47.8271	0.168009
4	newly 3 weeks spec	134.	252.39	{154., 348.}	88.4851	0.25542	newly 3 weeks cumul	346.	640.68	{417., 850.}	85.1676	0.251997	newly 5 weeks spec	273.	323.49	{199., 462.}	33.1685	0.12661	newly 5 weeks cumul	805.	1253.74	{788., 1712.}	57.4161	0.182781	newly 61 weeks cumul	4621.	6353.83	{5231., 7278.}	37.6116	0.135118
5	newly 3 weeks spec	76.	153.32	{134., 172.}	101.737	0.302845	newly 3 weeks cumul	221.	505.14	{484., 527.}	128.57	0.358696	newly 5 weeks spec	121.	122.58	{107., 139.}	8.28099	0.0356905	newly 5 weeks cumul	442.	764.18	{727., 800.}	72.8914	0.237479	newly 41 weeks cumul	2815.	1627.65	{1517., 1732.}	42.1794	0.238535
6	newly 3 weeks spec	89.	426.5	{58., 1207.}	392.045	0.541144	newly 3 weeks cumul	165.	836.31	{145., 2006.}	413.679	0.562256	newly 5 weeks spec	215.	631.43	{80., 1437.}	219.753	0.45565	newly 5 weeks cumul	499.	2030.77	{270., 5255.}	321.645	0.510856	newly 67 weeks cumul	3503.	7334.24	{4483., 9211.}	109.397	0.307112
7	newly 3 weeks spec	131.	227.11	{98., 382.}	83.458	0.230233	newly 3 weeks cumul	306.	559.58	{269., 948.}	88.1438	0.237441	newly 5 weeks spec	265.	308.7	{133., 556.}	50.2189	0.194522	newly 5 weeks cumul	790.	1137.	{521., 1924.}	61.281	0.193601	newly 63 weeks cumul	3676.	6240.83	{4863., 8087.}	69.7723	0.221867
8	newly 3 weeks spec	120.	255.66	{202., 298.}	113.05	0.321499	newly 3 weeks cumul	269.	680.93	{565., 783.}	153.134	0.397647	newly 5 weeks spec	198.	296.37	{237., 350.}	49.6818	0.167983	newly 5 weeks cumul	652.	1253.86	{1039., 1446.}	92.3098	0.27815	newly 58 weeks cumul	4528.	5748.48	{5240., 6312.}	26.9541	0.102412
9	newly 3 weeks spec	122.	243.4	{137., 364.}	99.7705	0.268994	newly 3 weeks cumul	305.	600.2	{369., 915.}	96.7869	0.26859	newly 5 weeks spec	202.	328.96	{181., 513.}	68.7327	0.205433	newly 5 weeks cumul	662.	1218.22	{717., 1855.}	84.852	0.239653	newly 62 weeks cumul	4588.	6603.54	{5397., 7758.}	43.9455	0.154122
10	newly 3 weeks spec	146.	265.08	{196., 355.}	81.6712	0.245955	newly 3 weeks cumul	372.	705.33	{552., 942.}	89.6048	0.266261	newly 5 weeks spec	191.	306.67	{222., 415.}	60.8848	0.19514	newly 5 weeks cumul	713.	1300.62	{973., 1724.}	82.4151	0.249642	newly 59 weeks cumul	3268.	5709.25	{4987., 6577.}	74.7017	0.239916
11	newly 3 weeks spec	101.	308.99	{123., 615.}	208.386	0.410928	newly 3 weeks cumul	225.	717.69	{298., 1383.}	219.827	0.435004	newly 5 weeks spec	200.	437.77	{168., 852.}	126.815	0.298043	newly 5 weeks cumul	597.	1537.44	{590., 2907.}	161.035	0.350933	newly 64 weeks cumul	2927.	6931.66	{4957., 8801.}	136.818	0.364901
12	newly 3 weeks spec	49.	270.43	{58., 623.}	453.857	0.603894	newly 3 weeks cumul	71.	579.38	{144., 1230.}	716.028	0.790904	newly 5 weeks spec	277.	420.26	{65., 941.}	93.9856	0.331683	newly 5 weeks cumul	466.	1359.29	{283., 3147.}	206.358	0.411437	newly 66 weeks cumul	3893.	6669.85	{3459., 8848.}	76.1123	0.234443
13	newly 3 weeks spec	133.	239.14	{187., 294.}	79.8045	0.248802	newly 3 weeks cumul	290.	637.77	{527., 782.}	119.921	0.337639	newly 5 weeks spec	295.	279.73	{218., 352.}	15.1559	0.0688672	newly 5 weeks cumul	831.	1178.01	{979., 1444.}	41.7581	0.146541	newly 58 weeks cumul	4651.	5645.05	{5205., 6253.}	21.3728	0.0829425
14	newly 3 weeks spec	178.	264.24	{201., 333.}	48.4494	0.1636	newly 3 weeks cumul	407.	686.2	{551., 848.}	68.5995	0.220297	newly 5 weeks spec	248.	320.83	{249., 406.}	30.2863	0.1071	newly 5 weeks cumul	867.	1302.04	{1054., 1628.}	50.1776	0.169249	newly 59 weeks cumul	4541.	6185.51	{5506., 6881.}	36.3094	0.132659
15	newly 3 weeks spec	89.	246.27	{157., 333.}	176.708	0.422888	newly 3 weeks cumul	247.	623.5	{421., 859.}	152.429	0.385585	newly 5 weeks spec	149.	325.64	{186., 445.}	118.779	0.318684	newly 5 weeks cumul	522.	1237.53	{793., 1680.}	137.075	0.356226	newly 61 weeks cumul	4384.	6408.04	{5210., 7342.}	46.1688	0.161007
16	newly 3 weeks spec	118.	202.92	{103., 298.}	76.8983	0.211795	newly 3 weeks cumul	242.	510.96	{282., 742.}	111.248	0.285948	newly 5 weeks spec	266.	275.56	{134., 411.}	37.9023	0.162321	newly 5 weeks cumul	698.	1023.94	{524., 1445.}	56.9885	0.174606	newly 62 weeks cumul	4382.	6114.35	{4788., 7447.}	40.2901	0.141083
17	newly 3 weeks spec	32.	237.99	{177., 313.}	643.719	0.858607	newly 3 weeks cumul	75.	619.78	{471., 791.}	726.373	0.907221	newly 5 weeks spec	45.	288.19	{213., 366.}	540.422	0.795606	newly 5 weeks cumul	154.	1169.82	{867., 1455.}	659.623	0.870323	newly 59 weeks cumul	3428.	5997.27	{5406., 6769.}	74.9495	0.241301
18	newly 3 weeks spec	96.	257.74	{182., 364.}	168.479	0.413617	newly 3 weeks cumul	238.	673.86	{503., 911.}	183.134	0.438852	newly 5 weeks spec	159.	314.19	{218., 439.}	97.6038	0.280494	newly 5 weeks cumul	518.	1276.36	{928., 1775.}	146.402	0.377989	newly 59 weeks cumul	2823.	6076.98	{5466., 6925.}	115.267	0.331043
19	newly 3 weeks spec	250.	294.15	{164., 470.}	40.46	0.152293	newly 3 weeks cumul	538.	722.72	{424., 1126.}	45.8476	0.151928	newly 5 weeks spec	410.	392.11	{212., 648.}	33.9293	0.158452	newly 5 weeks cumul	1241.	1462.97	{848., 2367.}	39.4126	0.148188	newly 62 weeks cumul	4288.	6718.61	{5393., 7945.}	56.684	0.190387
20	newly 3 weeks spec	215.	207.85	{92., 375.}	44.4884	0.200507	newly 3 weeks cumul	419.	505.66	{247., 929.}	47.1456	0.16993	newly 5 weeks spec	303.	285.18	{115., 550.}	46.0264	0.216042	newly 5 weeks cumul	946.	1037.02	{469., 1867.}	44.8837	0.178472	newly 63 weeks cumul	4612.	6252.47	{4466., 8076.}	37.4291	0.130775
21	newly 3 weeks spec	171.	205.38	{105., 357.}	42.1754	0.152375	newly 3 weeks cumul	333.	500.76	{274., 835.}	57.3153	0.175351	newly 5 weeks spec	270.	289.74	{138., 507.}	41.8444	0.172397	newly 5 weeks cumul	859.	1039.38	{539., 1729.}	41.6601	0.149857	newly 63 weeks cumul	4235.	6314.53	{4853., 7578.}	49.1034	0.167103
22	newly 3 weeks spec	162.	240.12	{174., 305.}	48.6543	0.16258	newly 3 weeks cumul	330.	631.65	{490., 774.}	91.4091	0.273639	newly 5 weeks spec	235.	287.71	{215., 365.}	25.5277	0.0934409	newly 5 weeks cumul	771.	1186.61	{934., 1487.}	53.9053	0.178883	newly 59 weeks cumul	3786.	5994.87	{5254., 6560.}	58.3431	0.198238
23	newly 3 weeks spec	145.	226.58	{95., 412.}	72.8276	0.215408	newly 3 weeks cumul	243.	535.74	{243., 927.}	122.922	0.29946	newly 5 weeks spec	283.	330.19	{121., 653.}	54.1025	0.215267	newly 5 weeks cumul	754.	1146.56	{504., 2116.}	69.0106	0.209053	newly 64 weeks cumul	3569.	6524.4	{4830., 7987.}	82.8075	0.253624
24	newly 3 weeks spec	208.	240.05	{167., 338.}	26.6779	0.100435	newly 3 weeks cumul	535.	622.26	{438., 851.}	25.3607	0.0953744	newly 5 weeks spec	227.	303.25	{197., 414.}	38.5947	0.131448	newly 5 weeks cumul	960.	1197.47	{833., 1654.}	31.0323	0.110679	newly 60 weeks cumul	2818.	6150.84	{5099., 7166.}	118.27	0.335576
25	newly 3 weeks spec	8.	365.5	{59., 794.}	4468.75	1.45097	newly 3 weeks cumul	23.	730.7	{125., 1541.}	3076.96	1.32654	newly 5 weeks spec	113.	578.17	{85., 1259.}	421.336	0.591799	newly 5 weeks cumul	209.	1797.73	{307., 3850.}	762.053	0.769641	newly 67 weeks cumul	3078.	7233.59	{4564., 9323.}	135.637	0.356465
26	newly 3 weeks spec	173.	288.09	{219., 388.}	66.526	0.211534	newly 3 weeks cumul	463.	769.43	{609., 1012.}	66.1836	0.211578	newly 5 weeks spec	234.	336.06	{257., 444.}	44.3077	0.150205	newly 5 weeks cumul	924.	1421.33	{1125., 1889.}	53.8387	0.177951	newly 58 weeks cumul	3097.	6078.71	{5385., 6753.}	96.2774	0.291244
27	newly 3 weeks spec	144.	232.07	{163., 319.}	61.1597	0.19054	newly 3 weeks cumul	310.	601.48	{439., 860.}	94.0258	0.274209	newly 5 weeks spec	330.	286.48	{201., 385.}	23.8545	0.116175	newly 5 weeks cumul	867.	1146.27	{818., 1599.}	34.248	0.116176	newly 60 weeks cumul	4073.	6000.15	{5083., 6874.}	47.3152	0.165318
28	newly 3 weeks spec	96.	100.3	{86., 115.}	9.91667	0.0411224	newly 3 weeks cumul	277.	337.52	{315., 365.}	21.8484	0.0846295	newly 5 weeks spec	113.	78.05	{65., 95.}	31.0177	0.166381	newly 5 weeks cumul	500.	501.74	{466., 531.}	5.28	0.0222877	newly 38 weeks cumul	2478.	1032.17	{926., 1161.}	58.3467	0.38208
29	newly 3 weeks spec	215.	227.33	{139., 323.}	25.7349	0.108012	newly 3 weeks cumul	511.	576.37	{385., 787.}	25.501	0.0998985	newly 5 weeks spec	312.	292.7	{177., 411.}	25.2372	0.119924	newly 5 weeks cumul	1054.	1131.68	{746., 1601.}	24.7211	0.101948	newly 61 weeks cumul	4420.	6300.53	{5321., 7250.}	42.5459	0.150897
30	newly 3 weeks spec	272.	236.32	{149., 341.}	25.2353	0.127296	newly 3 weeks cumul	607.	617.83	{413., 882.}	22.9868	0.0992573	newly 5 weeks spec	287.	295.52	{186., 429.}	26.6272	0.115076	newly 5 weeks cumul	1151.	1182.05	{761., 1685.}	24.2615	0.104163	newly 60 weeks cumul	4309.	5958.47	{4935., 7062.}	38.392	0.136953
31	newly 3 weeks spec	58.	346.64	{61., 756.}	503.034	0.605068	newly 3 weeks cumul	93.	702.42	{142., 1572.}	657.118	0.717938	newly 5 weeks spec	156.	538.29	{84., 1183.}	265.545	0.48625	newly 5 weeks cumul	351.	1704.46	{307., 3817.}	393.846	0.555911	newly 67 weeks cumul	3019.	7184.19	{4315., 9114.}	138.6	0.362865
32	newly 3 weeks spec	113.	144.12	{126., 165.}	27.5398	0.103346	newly 3 weeks cumul	325.	486.66	{451., 528.}	49.7415	0.174537	newly 5 weeks spec	137.	116.57	{97., 134.}	15.4234	0.0754785	newly 5 weeks cumul	581.	733.49	{683., 794.}	26.2461	0.100431	newly 40 weeks cumul	3452.	1521.73	{1382., 1663.}	55.9174	0.356866
33	newly 3 weeks spec	152.	233.53	{143., 343.}	56.5724	0.174554	newly 3 weeks cumul	297.	592.92	{381., 867.}	99.6364	0.277954	newly 5 weeks spec	297.	302.07	{176., 455.}	29.835	0.130057	newly 5 weeks cumul	807.	1165.41	{696., 1714.}	49.3371	0.158135	newly 61 weeks cumul	4655.	6359.46	{5436., 7372.}	36.6157	0.132518
34	newly 3 weeks spec	162.	230.32	{145., 332.}	46.716	0.152699	newly 3 weeks cumul	346.	591.06	{380., 816.}	71.1214	0.215521	newly 5 weeks spec	262.	293.45	{185., 422.}	29.0191	0.112947	newly 5 weeks cumul	775.	1146.82	{749., 1640.}	50.111	0.160884	newly 61 weeks cumul	4348.	6092.52	{5149., 7202.}	40.1826	0.143074
35	newly 3 weeks spec	234.	258.01	{161., 371.}	28.3889	0.112077	newly 3 weeks cumul	519.	655.11	{430., 940.}	34.1985	0.119235	newly 5 weeks spec	339.	332.42	{206., 494.}	26.0059	0.11629	newly 5 weeks cumul	1155.	1285.16	{829., 1828.}	28.0848	0.109462	newly 61 weeks cumul	4450.	6467.74	{5250., 7356.}	45.3425	0.158716
36	newly 3 weeks spec	156.	209.34	{95., 388.}	55.6667	0.178743	newly 3 weeks cumul	432.	509.18	{248., 920.}	43.8657	0.157393	newly 5 weeks spec	336.	293.69	{123., 574.}	45.9256	0.229224	newly 5 weeks cumul	978.	1053.68	{493., 1898.}	44.1759	0.176371	newly 63 weeks cumul	4342.	6374.09	{4835., 8105.}	47.4088	0.161699
37	newly 3 weeks spec	100.	330.67	{60., 867.}	246.91	0.424412	newly 3 weeks cumul	185.	700.22	{172., 1663.}	283.232	0.456308	newly 5 weeks spec	184.	493.41	{86., 1129.}	195.505	0.412147	newly 5 weeks cumul	548.	1622.72	{327., 4004.}	211.832	0.396779	newly 66 weeks cumul	3080.	6737.01	{3703., 9243.}	119.23	0.319488
38	newly 3 weeks spec	166.	237.22	{134., 336.}	49.5663	0.164227	newly 3 weeks cumul	393.	600.57	{353., 845.}	56.0025	0.179873	newly 5 weeks spec	256.	322.95	{168., 466.}	39.418	0.144567	newly 5 weeks cumul	843.	1205.95	{681., 1712.}	49.2325	0.164347	newly 62 weeks cumul	4770.	6506.74	{5283., 7603.}	36.4512	0.13089
39	newly 3 weeks spec	121.	228.23	{97., 397.}	95.0992	0.246778	newly 3 weeks cumul	299.	548.15	{254., 897.}	87.8294	0.235377	newly 5 weeks spec	257.	320.9	{136., 552.}	51.3541	0.182663	newly 5 weeks cumul	736.	1145.24	{505., 1928.}	67.2228	0.196599	newly 63 weeks cumul	3570.	6491.64	{4764., 8198.}	81.8387	0.251651
40	newly 3 weeks spec	88.	84.38	{70., 94.}	9.22727	0.042298	newly 3 weeks cumul	286.	288.5	{271., 304.}	3.8042	0.0163583	newly 5 weeks spec	121.	65.04	{55., 76.}	46.2479	0.273601	newly 5 weeks cumul	519.	428.14	{404., 453.}	17.5067	0.0840197	newly 36 weeks cumul	2280.	872.59	{772., 950.}	61.7285	0.418298
41	newly 3 weeks spec	60.	339.29	{58., 720.}	470.283	0.562644	newly 3 weeks cumul	169.	685.11	{147., 1501.}	312.16	0.46811	newly 5 weeks spec	165.	510.54	{86., 1258.}	228.388	0.427104	newly 5 weeks cumul	429.	1641.01	{297., 3640.}	292.096	0.461329	newly 67 weeks cumul	3396.	7106.92	{4062., 9077.}	110.126	0.306159
42	newly 3 weeks spec	170.	229.28	{162., 316.}	38.0588	0.128634	newly 3 weeks cumul	363.	610.37	{457., 817.}	68.1736	0.211889	newly 5 weeks spec	211.	270.18	{197., 380.}	32.019	0.109778	newly 5 weeks cumul	769.	1132.66	{829., 1519.}	47.8283	0.156124	newly 59 weeks cumul	3469.	5572.7	{4698., 6547.}	60.6428	0.202768
43	newly 3 weeks spec	65.	223.69	{63., 520.}	249.8	0.447493	newly 3 weeks cumul	145.	504.92	{159., 1109.}	252.028	0.461248	newly 5 weeks spec	321.	346.57	{76., 829.}	64.4081	0.305105	newly 5 weeks cumul	595.	1141.3	{304., 2632.}	114.38	0.304354	newly 65 weeks cumul	3665.	6580.61	{3683., 8666.}	82.3533	0.248134
44	newly 3 weeks spec	156.	238.56	{154., 320.}	55.0641	0.174732	newly 3 weeks cumul	333.	605.52	{404., 786.}	81.958	0.243613	newly 5 weeks spec	232.	306.05	{197., 437.}	39.4095	0.135105	newly 5 weeks cumul	768.	1184.43	{793., 1599.}	55.8112	0.177562	newly 61 weeks cumul	4274.	6256.27	{5252., 7252.}	46.3797	0.16224
45	newly 3 weeks spec	48.	291.73	{16., 705.}	534.854	0.680713	newly 3 weeks cumul	71.	567.5	{40., 1266.}	717.155	0.745758	newly 5 weeks spec	143.	499.58	{23., 1245.}	286.559	0.586284	newly 5 weeks cumul	285.	1481.86	{111., 3734.}	446.681	0.641544	newly 68 weeks cumul	4420.	6641.15	{2433., 8880.}	67.4572	0.264453
46	newly 3 weeks spec	89.	251.09	{56., 598.}	199.404	0.366677	newly 3 weeks cumul	211.	559.49	{146., 1244.}	177.673	0.345888	newly 5 weeks spec	199.	368.8	{72., 918.}	127.889	0.360822	newly 5 weeks cumul	585.	1246.45	{297., 3005.}	142.053	0.335268	newly 65 weeks cumul	3593.	6485.02	{3502., 8830.}	84.054	0.249781
47	newly 3 weeks spec	73.	278.71	{96., 587.}	281.795	0.500991	newly 3 weeks cumul	110.	620.16	{246., 1238.}	463.782	0.685773	newly 5 weeks spec	151.	435.16	{156., 896.}	190.278	0.387385	newly 5 weeks cumul	352.	1416.54	{513., 2864.}	302.426	0.530719	newly 65 weeks cumul	4475.	7220.14	{5216., 9012.}	61.8547	0.20102
48	newly 3 weeks spec	120.	241.86	{127., 358.}	101.95	0.273435	newly 3 weeks cumul	221.	600.68	{347., 832.}	171.801	0.408655	newly 5 weeks spec	260.	327.78	{174., 490.}	41.8	0.149438	newly 5 weeks cumul	652.	1216.76	{693., 1757.}	87.4172	0.245893	newly 62 weeks cumul	3589.	6611.44	{5474., 7538.}	84.214	0.261404
49	newly 3 weeks spec	81.	274.49	{200., 369.}	238.877	0.51862	newly 3 weeks cumul	266.	740.44	{586., 964.}	178.361	0.435615	newly 5 weeks spec	116.	319.71	{244., 419.}	175.612	0.430031	newly 5 weeks cumul	474.	1360.14	{1060., 1778.}	186.949	0.448817	newly 58 weeks cumul	3702.	5821.94	{5164., 6595.}	57.2647	0.194643
50	newly 3 weeks spec	137.	310.63	{223., 431.}	126.737	0.341986	newly 3 weeks cumul	367.	832.98	{593., 1127.}	126.97	0.343999	newly 5 weeks spec	235.	355.41	{258., 464.}	51.6723	0.170144	newly 5 weeks cumul	808.	1522.73	{1118., 2068.}	88.4567	0.263899	newly 58 weeks cumul	2666.	5985.51	{5160., 6884.}	124.513	0.349
